# Managing laboratory waste from HIV-related molecular testing: Lessons learned from African countries

**DOI:** 10.1016/j.hazl.2021.100030

**Published:** 2021-11

**Authors:** Collins Otieno Odhiambo, Anafi Mataka, Getachew Kassa, Pascale Ondoa

**Affiliations:** a African Society for Laboratory Medicine, Addis Ababa, Ethiopia; b ICAP at Columbia University, New York, United States; c Amsterdam Institute for Global Health and Development, University of Amsterdam, Department of Global Health, Amsterdam, the Netherlands

**Keywords:** Laboratory waste management, Viral load, LabCoP, Guanidinium thiocyanate

## Abstract

Waste generated from HIV viral load (VL) testing contains potentially hazardous guanidinium thiocyanate (GTC). GTC is toxic to humans and can pollute waters and harm aquatic life if not disposed of appropriately. We assessed gaps in waste management (WM) policies, regulations and practices through a self-assessment scorecard and an online survey questionnaire among 11 African countries participating in a laboratory systems strengthening community of practice and receiving technical assistance to scale-up VL testing. We identified solutions from national stakeholders, technical agencies, and manufacturers to inform interventions for improving WM. Nine of 11 countries did not have WM policies/guidelines in place. Most Countries reported disposing liquid chemical waste into the sewer. Nine countries prioritised the development of policies as a multi-sectoral approach in the short term. High-temperature incineration through cement factory kilns was identified as an effective, inexpensive and high-capacity disposal option for GTC-containing waste in the short term. A long-term consideration with funding from governments and donors were infrastructural investments for conventional high-temperature incineration where cement factory kilns are unavailable/inaccessible. Adequate WM of GTC-containing waste through available funding could provide the necessary impetus to establish comprehensive WM systems addressing all types of healthcare waste through a multisectoral approach.

## 1 Introduction

Globally, waste generation is on the rise due to rapid population growth, urbanisation and industrialisation. It is estimated that by the year 2050, global waste will reach 3.4 billion tons, a 70 % increase from the 2016 level (The World Bank, 2019). Resource-limited countries are comparatively impacted more severely by unsustainably managed waste, resulting in serious health, safety, and environmental consequences. Healthcare waste includes all the waste generated by healthcare facilities, research institutions, and medical laboratories and is particularly concerning. About 10–25 % of healthcare waste is regarded as hazardous and may create various health risks within and outside the health facility (WHO publication, 2014).

The unprecedented global scale-up in HIV viral load (VL) and early infant diagnosis (EID) testing over the last decade in response to the HIV epidemic has contributed to the generation of large quantities of chemical healthcare waste comprising solid, liquid, and gaseous chemicals. Since 2013, the World Health Organization (WHO) recommended VL to monitor antiretroviral treatment, which has resulted in significant VL testing scale-up programs (WHO, 2013). By the end of 2020, more than 30 million HIV VL tests were estimated to be performed globally, generating approximately 924,000 L of effluent chemical waste and 2.1 million kg annually (Sleeman et al., 2018). The problem surrounding the waste management (WM) of HIV VL in laboratories located in resource-limited settings is growing in the context of increasing demand for VL testing. Exacerbating factors include the laboratory sector's overall limited resources and poor infrastructure, resulting in poor compliance to biosafety and biosecurity requirements, including waste management.

Molecular testing performed for HIV VL and EID produces mixed liquid chemical waste containing Guanidinium Thiocyanate (GTC), a corrosive and hazardous chemical capable of causing skin burns and eye damage and is harmful to human and aquatic life (Biospectra, 2021). GTC is part of many other molecular assays used in the extraction of DNA and RNA (Welch et al., 2020). Contact between GTC and oxidising agents like bleach, widely used in the laboratory setting for disinfection purposes, can lead to the release of toxic cyanide gas. The issue of WM is further compounded by the introduction of WHO pre-qualified Point of care (POC) and near POC testing technologies (such as HIV VL and EID on Cepheid GeneXpert platforms (WHO, 2016, 2017) in clinics and smaller laboratory structures that lack the infrastructure and adequate human resources to ensure proper management of GTC-containing waste (e.g., incinerators).

While the manufacturers of the HIV VL, EID and other molecular testing platforms recommend that GTC-containing liquid waste be disposed of according to country-specific regulations, guidelines, or policies, the reality is that WM may lay in a regulatory vacuum in many sub-Saharan African countries as many countries lack the relevant guidelines, or policies (Kahn Danielle et al., 2009). The WHO Blue Book (WHO publication, 2014) provides a good resource for countries that are yet to develop national regulations and policies addressing medical waste. Currently, it is unclear to what extent countries manage to translate the Blue Book guidance into actual, implementable policies that effectively support waste management.

Pragmatic interventions are needed to address the disposal of toxic laboratory waste in a context where molecular testing in general and HIV VL and EID, in particular, are anticipated to increase, as part of the most recent recommendations from global health agencies (WHO, 2013). Practical solutions have been described to address the issue of GTC-containing waste. For instance, WHO proposes high-temperature incineration, encapsulation and landfill, as solutions to manage GTC-containing liquid waste. In addition, some countries or experts may have identified or piloted good ideas to tackle waste disposal in resource-limited settings like the use of cement factory kilns and outsourcing of WM to private companies, which may potentially be reproduced or scaled up in similar settings.

Against this background, we mapped out the availability of WM policies and practices in 11 Sub-Saharan countries participating in the laboratory systems strengthening community of practice (LabCoP) and receiving technical assistance to scale up their HIV VL testing (Ondoa, 2019). We also set out to identify feasible solutions from national stakeholders, technical agencies and manufacturers that can inform future interventions to improve the systems for the GTC-WM in participating countries. The results reported here provide unique perspectives to countries, programs and funding organisations on the current gaps and opportunities to improve laboratory WM systems in low resource settings of Africa.

## 2 Materials and methods

### 2.1 The LabCoP

The LabCoP, launched in 2017, is a community of practice convened by the African Society for Laboratory Medicine (ASLM) and funded by the Bill and Melinda Gates Foundation (Ondoa, 2019). It aims to support joint knowledge creation and the quick adoption of ideas proven to work, to accelerate the scale-up of VL testing among sub-Saharan African countries. The LabCoP is composed of multi-disciplinary teams of national stakeholders at the central level, gathering clinicians, laboratorians, civil society, implementing partners and representatives of people living with HIV, all nominated by the ministries of health. Eleven countries (Democratic Republic of Congo, Ethiopia, Kenya, Malawi, Sierra Leone, South Africa, South Sudan, Tanzania, Uganda, Zambia, Zimbabwe) signed up to LabCoP at the inception of the project. The project’s theory of action proposes to translate good practices and knowledge generated through structured discussion, face-to-face meetings and country visits into ‘fundable’ action plans. LabCoP helps country teams to select interventions addressing the areas of weaknesses identified through the systematic assessments of each step of the HIV VL testing cascade (from test request to test result utilisation) (Ondoa, 2019) and of the supporting systems (including policy framework, biosafety & biosecurity, quality management and human resources).

### 2.2 Country self-assessment of national HIV viral load testing cascade

Recognizing the lack of a matrix to evaluate system gaps along each steps of the national HIV testing cascades, the ASLM team, assisted by ICAP at Columbia university and stakeholders from the LabCoP oversight committee (including: Office of the Global AIDS Coordinator and

Health Diplomacy, International Laboratory Branch, Division of Global HIV and TB at US Centers for Diseases Control (US CDC), International Treatment Preparedness Coalition, Bill & Melinda Gates Foundation, Médecins Sans Frontières, Africa Centers for Disease Control and WHO) designed a scorecard complementary to existing tool applicable to the laboratory facility level (ASLM, 2021a). The LabCoP HIV viral load self-assessment scorecard is based on the capability Maturation Model (Team, C.P., 2010), It uses a scoring system from 1 to 4, with each component assigned the lowest score given to any question within that component, highlighting weaknesses. The domains covered by the scorecard include demand creation for HIV VL testing, specimen collection and processing, sample transportation, HIV VL testing, WM and biosafety, results utilization and leadership and management. The scorecard was validated by the 11 LabCoP country teams prior to its application. In order to mitigate the country ‘assessment’ fatigue and to promote ownership of the evaluation and improvement process, the tool was designed to be used for self-assessement by the LabCoP multidisciplinary country teams composed of at least 5–15 members which include national designees in charge of various aspects of the viral load testing cascade in each country drawn from clinicians, laboratorians, civil society and implementing partners among others. Country team members completed the scorecard together following a standardized procedure provided in the form of a user guide, with answers to the questions representing the consensus agreement of the team. The scores under each domain were collated across countries to identify areas with the lowest cumulative scores.

### 2.3 Survey of waste management practices

**Fig. 1:**
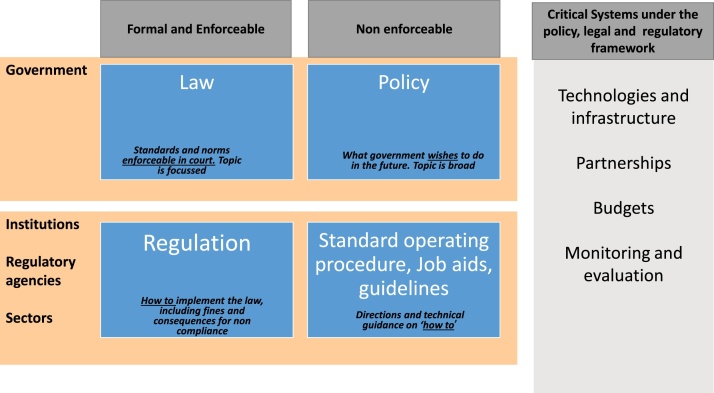
Legal and regulatory framework for WM.

In order to obtain more insight into the reported weaknesses in the WM area, the data from the self-assessment exercises were complemented with a survey conducted between November 2018 and March 2019 (Supplementary material 1), seeking to determine the availability of national WM policies, laws and regulations. A detailed definition of laws, policies, regulations and guidelines is given in Fig. 1. The survey was conducted through an online questionnaire, which also explored the presence of WM regulation bodies that support the implementation of the policy through law enforcement and the availability of treatment technologies for hazardous waste, the waste categories and the disposal practices for VL liquid waste at testing facilities. The responses to the survey questionnaire were shared by the LabCoP country leads and represented the consensus agreement of the 5–15 country team members. The responses were summarized into proportion for each question against the number of respondent countries.

### 2.4 Identifying waste management strategic options through structured discussions

We explored and documented country experiences and the roles and perspectives of manufacturers through a sub-community of practice (sub-CoP) dedicated to WM and launched in March 2019. The WM sub-CoP targeted WM stakeholders nominated by respective countries. The goal of the WM sub-CoP was to exchange knowledge and experience through structured discussion articulated around 3 topics: (1) theoretical training based on the WHO blue book; (2) exchange and documentation of WM solution involving manufacturers of *in vitro* diagnostic products and subject matter experts; (3) assisting country teams in designing targeted interventions based on the country’s self-assessment results complemented by dedicated surveys. The interactions took place through the monthly extension for community health outcomes (ECHO) (Project ECHO, 2021) and one face-to-face symposium as part of the annual LabCoP meeting in Addis Ababa, Ethiopia, in October 2019. The ECHO sessions were recorded and archived (ASLM, 2021b) with questions and answers addressed during the sessions through the chat boxes for later thematic analysis across the following pre-defined cross-cutting issues: policy and regulatory framework, financing, standard procedures and practices, monitoring and evaluation, infrastructure, and use of innovative technologies. Most frequent themes mentioned through the discussions were identified through coding of transcripts using the method described by Lewinski et al. (2019) and summarized as strategic options under corresponding pre-defined cross cutting topics. The strategic options were validated and refined by country teams and stakeholder through one round of online feedback. All interventions reported by participants as part of the didactic presentations or the discussions were inventoried and categorized according to strategic areas by two ASLM researchers, after consensus.

### 2.5 Statistical analysis

For identification of domains of the HIV self-assessment tool, with the greatest weakness, we assigned each component the lowest score given to any question within that component. The scoring ranged from ‘1’ representing poorly defined processes to ‘4’ corresponding to well defined processes and continuously improved. We collated the scores within each domain across the countries to identify the domains with the greatest weakness represented by the lowest cumulative scores. The data were analyzed using Power BI. Data from the survey questionnaire were expressed as proportions against the number of respondent countries.

## 3 Results

### 3.1 Waste Management is as a priority area within the LabCoP countries

The results from the country self-assessments highlighted that the laboratory WM domain was a common gap affecting the biosafety around the scale-up of VL testing and had the lowest cumulative score of 23 (range: 23–37) across the 8 domains in the 11 countries (Table 1). From the self-assessment exercise at baseline, 10 of the 11 LabCoP countries identified gaps on the WM domain, with 9 of 11 countries reporting poorly defined/poorly documented processes for WM (Table 1) as well as the lack of policy and guidelines. Further analysis of countries' responses to individual questions under the WM domain reveals that seven countries were in the process of developing their WM policy/biosafety guidelines. Two countries did not have WM policy/biosafety guidelines in place. One country reported the existence of national WM policy/biosafety guidelines but estimated that these were generally not used in most VL testing laboratories.

**Table 1:** Baseline self-assessment scores of the laboratory system to support VL testing scale-up among LabCoP countries.

**Legend**: The final assessment per domain is allocated a score corresponding to the following 4-levels of increasing maturity according to the Capability Maturation Model. **1(RED)**-Poorly defined processes; **2 (YELLOW)**-Processes understood but poorly documented; **3 (LIGHT GREEN)**-Key processes documented and **4 (DARK GREEN)**-Processes well defined and continuously improved.

### 3.2 Gaps in policies and practices among individual LabCoP countries

Six countries that responded to the WM survey reported the generation of chemical, sharps, infectious, non-hazardous and general waste types at 11–20 VL testing facilities [ASLM, 2021c, data not shown]. Four of the 6 countries reported the existence of WM policies and regulations on treatment, storage and disposal of hazardous waste and the presence of a national enforcement body. Thermal technology for WM was also available in 5 of the 6 countries. However, 4 of the 6 countries reported disposal of HIV VL testing-related liquid waste by pouring down the drain despite the availability of thermal technology for WM. Although 5 LabCoP country teams did not respond to the survey, it is conceivable that they might be faced with similar gaps in WM practices.

### 3.3 Strategic options and priority actions to address waste management gaps as identified through the waste management sub-Community of Practice

Twelve ECHO/webinar sessions with approximately 30–90 participants per session were conducted between March 2019 and February 2020, covering available WM technologies, country experiences and manufacturer perspectives on WM (Table 2). Four countries, South Africa, Malawi, Mozambique (guest country) and Zimbabwe, shared details on their policy and regulatory framework and WM practices, which informs the feasibility of strategic options regarding policy and regulatory framework, infrastructure and the use of innovative technologies. The best solutions and most successful interventions contextualized during the webinars and identified during the ECHO sessions were all summarised by the LabCoP team into a decision-making matrix for the selection of appropriate WM interventions that can be used as a guide by countries lagging in implementation of appropriate WM interventions. The themes identified through the independent coding are summarized in Table 3.

**Table 2:** ASLM/CDC waste management impact training curriculum, March 2019 to March 2020.

Month	Presenter	Topic	What was done/achieved
Mar-19	Subject matter expert	Review of LabCOP Waste Management Questionnaire	Reviewed countries’ current practices based on survey feedback. Requested participation from in-country WM TWG or related group. In advance of session, from countries that responded affirmative to having hazardous waste management policies/guidelines/regulations in place, requested them to share with ASLM.
April-19	Subject Matter expert	WHO Healthcare Waste Management Blue Book: Safe management of wastes from healthcare activities.	Reviewed the current best practices and guidance from WHO on waste management (materials shared in previous session) and bullet points for liquid chemical waste.
May-19	Subject matter expert	Tool for Waste Management Considerations for Viral Load and Early Infant Diagnosis (EID) Testing Laboratories and Associated Healthcare Facilities	Introduced VL WM checklist tool (developed by ILB, ASLM, GF and partners) for baseline audit of select country VL labs (initially as part of meeting measurement for demonstrated improvement per work plan).
June-19	Country experience	Viral load waste management: Mozambique Field Experience.	Presented an Overview of the Pros/Cons of the various methods of WM treatment, to include relative costs associated, sustainability, etc.
July-19	Country experience	Development of National and Facility WM Policies	Using the South African example, presented Key steps in getting policies established at national level; personnel and communication needed; coordination with local contractors; stakeholders, who enforces, etc.
Aug-19	Subject matter expert	Update: Tool for Waste Management Considerations for Viral Load and Early Infant Diagnosis (EID) Testing Laboratories and Associated Healthcare Facilities	Presented an improved version of the tool after incorporating the feedback.
Sept-19	Subject Matter expert	Approaches for Waste Incineration	Incineration identified as one of the major current solution, the session presented an overview of the various options available
Oct-19	Manufacturer perspective	Perspectives of a Private Waste Management Practitioner	Experiences of innovative and sustainable medical waste management: perspectives of a Private Waste Management Practitioner
Nov-19	Manufacturer perspective	Manufacturer perspective: Abbott Laboratories	Abbott their experiences, expertise and advice on waste handling.
Jan-2020	Manufacturer perspective	Manufacturer perspective: Roche Diagnostics	Roche Diagnostics their experiences, expertise and advice on waste handling.
Feb-2020	Country experience	Kenya country team presentation on experience using WM assessment tool	Findings from the Assessment of Waste Management Practices in Viral load & EID laboratories in Kenya
Mar-2020	Manufacturer perspective	Manufacturer perspective: Hologic	PANTHER: Waste and Contamination Management

**Table 3:** Strategic decision matrix for improving WM practices.

Strategic areas	Strategic options	Priority action items for improvement
Policy and regulatory frameworks	• Ensure that national legal policy, and regulatory framework to address waste segregation, audit trail to waste destruction, and defined roles and responsibilities of stakeholders • Review legal, regulatory and policy framework to address GTC waste across all sectors • Improve practical guidance for WM at both national and facility level	• Develop standards for national WM, with support from LabCoP and other collaborating partners on the ground e.g., US CDC, MSF, CHAI, UNICEF etc. • Adapt regulatory or legal frameworks from other countries (e.g., Basel Convention signing as South Africa and Mozambique) • Include WM in national laboratory policy and strategic plan • Include guidance /SOPs on chemical waste management – examples from Malawi and Zimbabwe
Governance and coordination	• Establish technical working group (TWG) for national coordination • Assign roles and responsibility for WM at all levels of the laboratory system (central, regional, district, facility) • Involve manufacturers or suppliers in the disposal of waste (Apply reverse logistic	• Consider/advocate for a dedicated office of waste management and biosafety at national/regional, facility level on the South Africa and/or Kenya models. • Establishment of functional national TWG, including implementing partners and stakeholders (on the model of Kenya) • Leverage well-established system for transporting essential medicines and other medical supplies to health care facilities • Assign roles and responsibility to stakeholders e.g., Kenyan model where disposal of expired products (reagents, drugs) is the responsibility of the central supply agency • Design clear job descriptions and provide necessary training for WM at all levels using the entry point of the HIV program including a maintenance team to service incinerators using the ASLM/PEPFAR collaboration model.
Financing	• Leverage current funding opportunities from PEPFAR, the GF and and donors around HIV, TB, Malaria work • Request fund from manufacturers as part of their social duty • Obtain clear cost estimate of differentiated laboratory waste management as part of the overall testing services	• Review costs associated with WM (e.g., VL machine specific WM) e,g., using the Clinton health Access Initiative model used in Zimbabwe costing exercise. • Submit plans to GF programing or reprogramming cycle, PEPFAR COP, and others, and allocate money, as part of the LabCoP • Establish contact with private companies for an efficient way of handling waste e.g., public-private partnerships (LabCoP to facilitate follow up meeting and summarise efforts through a white paper)
Infrastructure	• Expand the implementation of onsite conventional systems for waste disposal • Leverage the utilization of cement factories for high temperature incineration • Install new systems and innovative strategies • Improve the transportation system for waste from place of generation to place of destruction	• Optimize number and location of incinerators for managing the waste including the VL waste - GTC extraction of DNA and RNA, using GIS based modelling (e.g Labmap, OptiDx) • Pilot systems such as drainage, or charcoal absorption, solvent recovery and immobilization options. • Transport of waste to local cement factory • design a cost-effective waste transportation system on the model of sample transport in Uganda or South Africa. • Develop standards for infrastructure (e.g., drainage and WM systems) • Comply with recommendations and standards of the WM system, and strict consideration in the design phase of new laboratories and renovation. This includes the complete flow of waste from generation to disposal areas (e.g., storage, treatment, and disposal). • Update inventory of essential spare parts for the range of incinerators in-country.
Partnerships and collaboration	• Collaboration between disease programs for cost sharing • Public private partnerships for transportation and disposal of waste • cross border collaboration • include WM cost as part of contractual agreements with manufacturers	• Include manufacturer take-back schemes into contract agreements • Select diagnostic technologies with less toxic chemical alternatives to GTC in diagnostic tests, e.g., Guanidine Hydrochloride, and Sodium Hypochlorite. • Manufacturers to conduct research and development on effective WM solutions • Manufacturers and donors install high temperature incinerators in low-to-middle income countries as part of their social responsibility and aligned with national policies. - Manufacturers revise cost per test according to the ‘polluter pays’ principle, where they share responsibility for the cost of WM of their products.
Monitoring and Evaluation	• Reduce opportunities for non-compliance to good WM practices at national and facility level • Monitor GTC containing waste from generation to processing to destruction • Assess the cost effectiveness of various methods of WM	• List and prioritize measurable indicators for monitoring WM against set standards (e.g ‘certificate of destruction’ used as proxy for waste destruction by NHLS, South Africa) • Implement use of waste calculator to quantify amount of waste generated by testing facilities (e.g., SAWIS used to monitor amount of waste generated monthly in South Africa) • Implement WM dashboard at facility and national level for ease of monitoring. • Design audit and assessment schedules using the South African model. • Compare the cost of various WM method in relevant set up (e.g., Zimbabwe, Malawi, Mozambique costing models) • Establish baseline, and introduce regular monitoring using standard checklists for example the GF capacity assessment for health care WM, or the tool for VL and EID molecular WM considerations

#### 3.3.1 Policy and regulatory framework

Strategic options supporting the availability of comprehensive policy, legal and regulatory framework defining roles and responsibilities at each level of the healthcare system were identified, with South Africa offering useful examples. More specifically, Mozambique and South Africa, were the only signatory countries to the Basel Convention hence can transport waste to other signatory countries for destruction (United Nations Enviroment Program, 2021). South Africa is the most advanced among the LabCoP countries with a comprehensive arsenal of WM laws, policies, regulations and guidelines at both national and facility levels (South Africa Waste information centre, 2021), describing roles and responsibilities at each level. All waste in South Africa is governed under the Waste Act 59 of 2008 supported by at least 11 other laws (South Africa Waste information centre, 2021). The National Policy on Thermal Treatment of General and Hazardous Waste of 2008 outlines the framework for thermal waste treatment, including high-temperature incineration and co-processing in cement factories (South Africa government, 2008). The national waste information regulations govern waste transportation and generation including monthly reporting of waste quantities. Norms and standards also exist to guide storage, assessment and disposal of waste. There are local regulations that include stiff penalties for non-compliance. At the facility level, the National Health Laboratory Services (NHLS), has drafted the SANS 10248: Management of Health Care Risk Waste from a Health Care facility (SANS 10248, 2021). On WM based on the South African national standards (SANS), which applies to a network of 350 laboratories providing laboratory and related public health services including VL testing and EID to over 80 % of the South African population In addition, an internal waste management policy in line with the SANS 10,248 standard outlines the minimum provisions for the safe and effective management of waste generated by the VL testing laboratories to reduce potential risks to humans and the environment.

WM in Malawi is governed by the Environmental Management Regulations 2008 and the Infection control and WM plan (policy) 2016 (Malawi Environmental Management Regulations, 2008; Malawi Waste management Plan, 2016). Mozambique has three WM regulations covering health care and hazardous waste management and published in 2003, 2006 and 2014 (ASLM, 2021d). No guidance applicable to local health authorities, health facilities of VL testing laboratories were reported by these countries.

#### 3.3.2 Governance and coordination

National coordination through a TWG and roles and responsibilities at each levels of the health systems, with a role for manufacturers, emerged as strategic option for improving the governance for WM. Useful examples such as establishing a dedicated office for WM and biosafety at the national level, forming a TWG including implementing partners and funders (Kenya) or leveraging the funding earmarked for the HIV programmes (PEPFAR recommendation) to deliver the necessary training, were mentioned as priority actions.

#### 3.3.3 Financing

The strategic option for financing waste management included leveraging on existing global funding earmarked for HIV programmes and engaging with manufacturer, through request supported by clear estimates of waste volumes at facility and national levels. PEPFAR, the Global Fund and other organizations. PEPFAR has included WM of GTC-containing waste as a priority in the Country Operational Plans (COP21) (PEPFAR, 2021). The Global Fund has also dedicated funding towards WM under its Resilient and Sustainable Systems for Health Modular Framework Intervention Package (The Global Fund, 2021). Manufacturers of diagnostic tests can also play a role in financing some of the WM activities as part of their social responsibility e.g. through public-private partnerships. Some priority action can take advantage of the US CDC/Roche Diagnostics partnership aiming at deploying waste calculator tools and instrument agnostic waste costing frameworks, which can be readily implemented in LabCoP countries and in collaboration with ASLM. Other priority actions include providing support to countries to apply for PEPFAR and GF funding opportunities. This will be an important component for making an investment case for funding towards sustainable WM practices.

#### 3.3.4 Infrastructure

One of the strategic options includes leveraging on existing in-country infrastructure. Cement factory kilns are an attractive alternative to high temperature incinerators as demonstrated through piloting in Mozambique and Malawi. Cement factory kilns allowed high-temperature incineration of larger quantities of hazardous waste, i.e., 1% of 200 tons per hour compared to 1% of 15 kg every 15 min for high-temperature incinerators (ASLM, 2021d). Ideally, one cement factory kiln had enough capacity to handle the estimated VL waste from these countries including other wastes including cytotoxic and pharmaceutical waste generated from the testing facilities. However, this alternative was not devoid of challenges, including long term hazardous waste storage and transport and multiple agreements required between cement factories and the relevant ministries and other stakeholders, including the ministry of environment, ministry of health and transport companies. LabCoP countries will conduct mapping exercise based on geographic information system information to locate functional incinerators and cement factory kilns to develop sustainable waste transportation systems.

#### 3.3.5 Partnerships and collaboration

One strategic option to address WM gaps is manufacturers of diagnostic tests employing innovative strategies to minimise the environmental impact of their products using less toxic chemical alternatives to GTC for nucleic acid extraction, e.g., Abbott mPima uses Guanidine Hydrochloride, and Panther VL tests use Sodium Hypochlorite (Anon, 2019; ASLM. Panther, 2021). Another strategic option is fostering take-back schemes by assuming responsibility for handling toxic waste generated by their equipment/tests. Manufacturers also need to share best practices on waste disposal at manufacturing sites in the form of the guidance document and practical resources. Lastly, manufacturers of test kits could consider revising the cost per test to include the cost of waste disposal. Collectively, the manufacturers’ responsibilities mentioned above, in addition to ongoing country’ efforts to address gaps in policies and practices, will go a long way in strengthening WM among LabCoP countries.

Cross collaboration between disease programs will lead to cost savings and efficiencies in managing waste products.

#### 3.3.6 Monitoring and evaluation

One strategic option identified is countries monitoring their waste from generation to destruction thereby reducing the opportunities for non-compliance to good WM practices at national and facility levels. Countries must prioritize selection of key measurable indicators for monitoring waste e.g., NHLS in South Africa monitors the return of waste destruction certificates to verify the destruction of waste by private waste disposal companies. South Africa also has a system to routinely estimate the amount of waste, through the South African Waste Information System, that captures routine data on the amount of waste generated, recycled and disposed of on a monthly and annual basis (SAWIC, 2021). Countries can adopt this system or estimate the amount of waste generated using the number of tests (Sleeman et al., 2018). Evaluations can also be employed to identify the most cost-effective WM treatment methods, for example Mozambique and Malawi were able to determine that leveraging on cement factory kilns was cheaper, costing about $1.0–1.52/kg compared to approximately $ 1.9–20/kg for conventional incineration (ASLM, 2021d).

## 4 Discussion

Our analysis of WM policies and practices among LabCoP countries confirms that disposal of GTC-containing waste is a priority in at least 9 of the 11 LabCoP countries, underscoring a larger problem with general chemical waste. Most of the LabCoP countries are still in the process of developing policies and regulations around WM for medical/chemical waste. The lack of regulations in place hampers the enforcement of proper WM practices within VL and EID testing facilities. Moreover, lack of WM standard operating procedures and job aids at these testing facilities lead to improper waste disposal practices like pouring liquid chemical waste down the drain. In addition, there is a lack of knowledge and/or capacity to systematically quantify the amount of waste generated from VL and EID testing facilities, including liquid chemical waste. Deducing from the estimates by Sleeman et al. (2018), the VL tests would translate to approximately 35,000 kg, 56,000 kg and 84,000 kg of solid waste and 15,000 L, 25,000 L and 37,000 L liquid chemical waste per annum in Malawi, Mozambique and Zimbabwe, respectively (Nyagadza et al., 2019; Ehrenkranz et al., 2019; Vubil et al., 2020). The amount of waste will increase with additional molecular COVID-19 diagnostic tests, which also contain GTC as testing is scaled-up in response to the pandemic. Thus, quantification and disposal of waste remains a systemic gap regardless of disease area and needs to be addressed to ensure proper WM to inform scale of intervention/mitigation strategy.

Some countries among the LabCoP have demonstrated good WM systems that can be conceptualised and reproduced in other countries to improve WM practices. South Africa, for example, has a strong national policy referring to international standards and linked to local regulations and robust monitoring and enforcement with hefty penalties for non-compliance. They also have a public private partnership for waste disposal and coordination of WM by a central authority with strong oversight through ad hoc audits and inspections. Proper WM in the testing laboratories is enabled by an internal WM policy and a strong laboratory governance structure through the NHLS.

In the short term, countries with gaps in WM must implement changes at their VL testing facilities to halt the disposal of liquid chemical waste down the drain. This will require revising guidelines, SOPs and job aids at the institutional level and procurement of containers for the temporary storage of liquid chemical waste before incineration. Countries must also conduct mapping exercises to determine the capacity and functionality of high-temperature incinerators both in the public and private sectors, including consideration of high temperature kilns at cement factories as an outsourcing alternative. Sustainability must be considered in selecting the most appropriate waste disposal method. Installation of high temperature incinerators requires a significant initial cost in addition to regular maintenance and constant supply of fuel to treat a limited volume of waste. On the contrary, the use of high-temperature cement kilns provides a more sustainable approach to WM as it allows the treatment of a significant volume of VL waste in addition to other types of waste, including cytotoxic and pharmaceutical waste. These WM interventions can be quickly implemented by countries through domestic financing or the PEPFAR country operational plans 2021 (COP21), the Global Fund, WHO, etc. or the intervention of other organisations. Traditional donor organisations have availed funding for WM. For example, WM of GTC-containing waste has been included as a priority area in the COP21 (PEPFAR, 2021). Under its Resilient and Sustainable Systems for Health Modular Framework Intervention Package, the Global Fund has dedicated funding for WM prioritising avoidance, reduction, and management of healthcare waste (The Global Fund, 2021). In the long term, policies and regulations must be formulated/revised to ensure compliance with the country’s WM standards.

The LabCoP and partners will continue to facilitate the dialogue with manufacturers on their commitments to improve WM and support the implementation of identified innovative WM methods. Such methods include absorption of liquid chemical waste using charcoal which is an attractive alternative as it reduces the volume of liquid chemical waste and is an accelerant during incineration. In addition, other methods such as chemical precipitation and neutralisation are being investigated. As large consumers of VL diagnostic tests, sub-Saharan African countries must concertedly engage manufacturer to include a WM component as part of the service support package offered to customers, ensuring that they acknowledge responsibility for the entire product lifecycle. Alternatively, take back schemes must be implemented in poor-resource settings where WM disposal technologies are unavailable.

One limitation of our study was the self-assessment method of data collection from the countries that may not accurately reflect the situation on the ground. However, in mitigation, we made follow up queries through the country LabCoP team leads and conducted desk reviews whenever information was available on the internet. Additionally, we shared the results of all countries during ECHO sessions, including the entire country teams and implementing partners.

## 5 Conclusions

This paper highlights the gaps and possible solutions in management of GTC-containing liquid waste and that this has become a priority in many African countries, as molecular testing is scaled up as part of large disease control programs and outbreak response. Even with considerations by manufacturers to take a significant role in product life management to include the final disposition of waste generated, the problem of healthcare-related waste management will remain for our foreseeable future. This calls for an increased commitment of domestic resources as part of a solid multi-sectoral policy and sustainable regulatory framework addressing WM. Addressing the adequate management of waste generated through HIV viral load testing and available funding from the PEPFAR and GF programs could provide the necessary drive to establish comprehensive systems, based on solid policy and regulatory frameworks ultimately addressing all types of healthcare waste through a multisectoral approach. Examples of solutions that work are available from some more advanced African countries. LabCoP will continue facilitating the expansion of solutions proven to work in similar settings.

## Ethics approval and consent to participate

The data from participant countries is part of programmatic data for the LabCoP and is publicly available. The country MOHs consented to share data on their viral load cascade.

## Funding

The LabCoP project is funded by the 10.13039/100000865Bill and Melinda Gates Foundation (grant number OPP1162196). Unitaid also funded part of the LabCoP activities through the Accelerating access and integration of innovative point of care technologies in diagnostic programs grant. Other funding support came from CDC PEPFAR Grant no. PAC/20190912/03. The WHO and Global Fund co-funded the LabCoP 2019 annual face-to-face meeting in Addis Ababa, Ethiopia. The funders had no role in the publication of this manuscript.

## Authors' contributions

Conceived the idea: PO. Collected data: AM, CO, KG. Analysed the data: PO, CO, AM, KG. Wrote the manuscript: CO, PO. Reviewed and approved the final manuscript: CO, PO, AM, KG.

## Declaration of Competing Interest

The authors report no declarations of interest.
