# Supplementary material for: Managing laboratory waste from HIV-related molecular testing: Lessons learned from African countries
Source: J Hazard Mater Lett. 2021 Nov;2:None. doi: 10.1016/j.hazl.2021.100030 (PMC8721957; doi:10.1016/j.hazl.2021.100030)
Supplement: Supplementary file 1 [file mmc1.pdf]

Country Specific Waste Management Survey

LIST OF QUESTIONS

1. What types of waste are collected, transported, and disposed at PEPFAR- supported testing facilities? Circle all that apply.

   a) Chemical waste: (e.g., waste that includes laboratory solvents and reagents, disinfectants, acids, bases, flammable liquids, used for
   b) nucleic acids extraction or preservation (e.g., formalin, formaldehyde, paraformaldehyde, alcohol, etc.), etc.)
   c) Cytotoxic waste: (i.e. highly hazardous substances that are, mutagenic, teratogenic or carcinogenic, such as cytotoxic drugs used in
   d) cancer treatment and their metabolites)
   e) Infectious waste: (e.g., waste contaminated with blood and other bodily fluids, cultures or stocks of infectious agents, disposable
   f) medical devices, testing cartridges and kits contaminated with infectious material, etc.)
   g) Non-hazardous or general waste: (e.g., waste that does not pose any particular biological, chemical, radioactive or physical hazard)
   h) Pathological waste: (e.g., waste that includes human tissues, organs or fluids, body parts and contaminated animal carcasses)
   i) Pharmaceutical waste: (e.g., expired, unused and contaminated drugs and vaccines)
   j) Radioactive waste: (e.g., products contaminated by radioisotopes including radioactive diagnostic material or radiotherapeutic
   k) materials)
   l) Sharps waste: (e.g., syringes, needles, disposable scalpels and blades, etc.)
   Other: ___________________________

2. What treatment technologies for hazardous waste are currently available at the PEPFAR-supported testing facilities?
(Circle all that apply)

   a) Burn pits
   b) Chemical Treatment (e.g. use of chemical disinfection)
   c) Dry-heat treatment (e.g., use of hot-air ovens)
   d) Encapsulation (the process of filling containers with waste, adding an immobilizing material, and sealing)
   e) Microwave treatment (a steam-based process where treatment occurs through the action of moist heat and steam generated by microwave
   f) energy)
   g) Steam Treatment (e.g., use of autoclaves)
   h) Thermal treatment (the use of high temperatures in the treatment of waste such incinerator, pyrolysis, rotary kiln, etc.)
   i) Other: ____

3. How many viral load testing facilities are PEPFAR-supported?

   a) None
   b) 1 to 5
   c) 6 to 10
   d) 11 to 20
   e) 21 to 50
   f) More than 50

4. How is liquid chemical waste from the VL testing platforms currently being disposed at these PEPFAR-supported testing facilities?

a) Encapsulation (i.e., filling containers with liquid waste, adding an immobilizing material, and sealing)
b) Poured down the sink
c) Thermal treatment (e.g. Incineration)
d) Don’t Know
e) Not applicable

Other Method: ________________________

5. Are there national regulations and policies that regulate the treatment, storage, and disposal of hazardous waste?

a) Yes
b) No
c) Don’t know

6. Are there regulatory bodies that enforce these national regulations and policies for the treatment,
storage and disposal of hazardous waste?

a) Yes
b) No
c) Don’t know
